# Seroprevalence and associated risk factors of hepatitis B virus among pregnant women in southern Ethiopia: a hospital-based cross-sectional study

**DOI:** 10.4178/epih.e2016027

**Published:** 2016-06-19

**Authors:** Yeshi Metaferia, Walelign Dessie, Ibrahim Ali, Anteneh Amsalu

**Affiliations:** 1 Department of Medical Laboratory Sciences, College of Medicine and Health Sciences, Wollo University, Dessie, Ethiopia; 2 Department of Medical Laboratory Sciences, School of Allied Sciences, College of Health Sciences, Addis Ababa University, Addis Ababa, Ethiopia; 3 Department of Medical Microbiology, College of Medicine and Health Sciences, School of Biomedical and Laboratory Sciences, University of Gondar, Gondar, Ethiopia

**Keywords:** Hepatitis B virus, Pregnant women, Enzyme-linked immunosorbent assay, Ethiopia

## Abstract

**OBJECTIVES:**

Hepatitis B virus (HBV) is a major blood-borne and sexually transmitted infectious agent that is a significant global public health issue. The aim of this study was to determine the seroprevalence and risk factors of HBV among pregnant women attending the antenatal clinic of the Hawassa University referral hospital in Ethiopia.

**METHODS:**

A cross-sectional study was conducted from April to May, 2015. A total of 269 consecutive pregnant women attending antenatal consultations were enrolled. Sociodemographic information and data regarding possible risk factors were collected using a structured questionnaire. Hepatitis B surface antigen (HBsAg) screening was performed using an enzyme-linked immunosorbent assay, and the data were analyzed.

**RESULTS:**

The overall seroprevalence of HBsAg among the 269 participants enrolled in the study was 7.8% (n=21). The prevalence of human immunodeficiency virus (HIV) infection was 5.2% (n=14), of whom two participants (14.2%) were also positive for HBsAg. Study participants with no formal education (odds ratio [OR], 3.68; 95% confidence interval [CI], 1.27 to 10.68; p<0.05) were more likely to be infected with HBV than those who had completed at least secondary school. Although HBsAg was detected more often in pregnant women who had multiple exposure factors (8.8%, n=13) than in pregnant women who had not experienced possible risk factors (4%, n=1), this difference was not statistically significant (OR, 2.33; 95%CI, 0.29 to 18.63).

**CONCLUSIONS:**

A high prevalence of HBV infection was detected in the study population. Neither the type of risk factors nor exposure to multiple risk factors was significantly associated with HBV infection. Hence, screening pregnant women regardless of risk factors and improving awareness of the transmission routes of HBV within this group may reduce the risk of HBV infections.

## INTRODUCTION

Hepatitis B virus (HBV) is a major blood-borne and sexually transmitted infectious agent, and represents a serious global public health problem. HBV is approximately 100 times more contagious than human immunodeficiency virus (HIV) and is found in diverse populations and subpopulations [1,2]. Globally, 350 million people are chronic carriers. Of these, one million of them are expected to suffer serious illness and death from cirrhosis and hepatocellular carcinoma (HCC) [3]. Africa has the second largest number of chronic carriers after Asia, and is considered a region of high endemicity (≥8%) [3]. Although it is difficult to assess the exact burden of HBV in Africa, between 56% and 98% of the adult population show evidence of past exposure to HBV infection, and the seroprevalence of hepatitis B surface antigen (HBsAg) has been estimated to range from 6% to 20% [4]. In Ethiopia, studies among pregnant women have shown moderate endemicity, with the prevalence of HBsAg positivity ranging from 3% to 6.1% [5-9].

HBV is transmitted through parenteral or mucosal exposure to infected blood and body fluids, usually either by a vertical or horizontal route early in life in highly endemic areas, resulting in a high rate of chronic infections [10]. The rates of new infections and acute disease are highest among adults, but chronic infection is more likely to occur in persons infected as infants or young children. Chronically infected persons have an increased lifetime risk for cirrhosis and HCC, and also serve as the main reservoir for continued HBV transmission due to its largely asymptomatic nature [2,3]. HBV infection during pregnancy is also associated with a high risk of maternal complications and has effects on both the mother and child [11]. It has been reported that 10% to 20% of HBsAg-positive pregnant women transmit the virus to their babies and that those who are positive for both HBsAg and HBV antigen have a nearly 100% chance of transmitting HBV to their newborns [12].

Previous studies in Ethiopia have demonstrated that important sources of HBV transmission include blood transfusion; tattooing; a history of surgery, unsafe injections, or abortions; multiple sexual partners; and traditional practices such as scarification, circumcision, and ear piercing [5-9]. Although the association between HIV and HBV has become less prominent in Africans, evidence has been found indicating that HIV makes HBV-related liver disease develop more quickly [13] and HIV/HBV co-infection has serious effects on both pregnant women and infants. A previous study among pregnant women in Bahir Dar city showed an HIV/HBV co-infection rate of 1.3% [8].

Preventing mother-to-child transmission of HBV is therefore fundamental for reducing the burden of the disease in sub-Saharan Africa, where it is endemic. An effective strategy for reducing the incidence of chronic infections is maternal screening combined with post-exposure prophylaxis consisting of HBV vaccination immediately after delivery in all infants born to HBsAg-positive mothers, jointly with immunoglobulin prophylaxis [14]. However, limited information has been published regarding the epidemiology of HBV among pregnant women in the southern part of Ethiopia. Despite its effects on both the mother and child, the routine screening of pregnant women for HBV is not practiced in many health facilities in Ethiopia. Moreover, universal immunization of infants against HBV is performed as part of the National Expanded Program on Immunization starting at six weeks. Hence, data regarding HBV in pregnant women are fundamental for health planners and caregivers in order to support evidence-based interventions. Therefore, the aim of this study was to assess the seroprevalence and risk factors of positive HBsAg status among pregnant women receiving care at the Hawassa University referral hospital antenatal care (ANC) clinic.

## MATERIALS AND METHODS

### Study design and area

A cross-sectional study was conducted at the Hawassa University referral hospital from April to May, 2015. The hospital is located in Hawassa, the capital city of the Southern Nations, Nationalities and Peoples’ Region, 275 km south from the capital of Ethiopia, Addis Ababa, and is a tertiary-level teaching hospital that provides health services to over 12 million inhabitants in the region. The hospital ANC clinic provides routine antenatal screening services, such as HIV testing, hemoglobin determination, blood group testing, rapid plasma reagin testing, and urinalysis (for glucose, proteins, and infection) for more than 20 pregnant women per day.

### Study subjects

A total of 269 consecutive pregnant women attending antenatal consultations at the ANC clinic of the Hawassa University referral hospital were enrolled during the study period, with a participation rate of 100%. Pregnant women who experienced bleeding and required urgent interventions were excluded from the study.

### Data collection

After obtaining informed written consent from each participant, sociodemographic information and data about potential risk factors were collected using a structured questionnaire by an experienced midwife nurse.

### Blood sample collection, transport, and processing

Approximately 5 mL of venous blood were collected from each pregnant woman. After clotting, serum was separated from the whole blood and rapid HBsAg testing was performed to deliver the result on the same day. The leftover serum samples were separated and stored at -20°C at Hawassa University referral hospital laboratory. After sample collection, frozen serum samples were transported to a regional blood bank using a cold box.

### Laboratory testing

All samples were tested for HBsAg using rapid diagnostic tests employing the principle of immunochromatography (SD Bioline, Yongin, Korea). The SD BioLine HBsAg One Step Test is a qualitative, solid phase, two-site sandwich immunoassay for the detection of HBsAg in serum or plasma. The membrane is precoated with anti-HBsAg antibodies on the test band region and anti-mouse antibodies on the control band region. During testing, the serum sample reacts with the dye conjugate (mouse anti-HBsAg antibody colloidal gold conjugate) that was coated on the test strip. The mixture then reacts with anti-HBsAg antibodies on the membrane via capillary action and generates a red band. The presence of two red bands indicates a positive result. One red band at the control line indicates a negative result, while the absence of a red band at the control line indicates an invalid result. For further confirmation, the stored serum samples were transported to a regional blood bank and tested using an enzyme-linked immunosorbent assay (ELISA) (Dialab GmbH, Wiener Neudorf, Austria) [15]. Routine HIV testing in Ethiopia is uniformly performed using the established national rapid testing algorithm: the Kehua Bioengineering (KHB) test kit (Shanghai, China) is used as a screening test, followed by the HIV1/2 STAT-PAK assay if positive. If the STAT-PAK and KHB results are discordant, the Unigold HIV test is used as a tiebreaker to determine the result.

### Quality assurance

The validity and completeness of the data were verified by the principal investigator daily. The performance of the rapid HBsAg test kit was evaluated using known positive and negative controls obtained from ELISA-tested blood donors. Furthermore, the formation of a colored band at the control line acted as a procedural control and further validated the results. Based on the instructions in the manufacturer kit, positive and negative controls were used for each ELISA reagent lot and positive results were repeated.

### Definition

The prevalence of HBV infections was determined using serologically positive results for HBsAg using the ELISA method.

### Data management and analysis

The data were coded, entered, cleaned, and analyzed using SPSS version 20.0 (IBM Corp., Armonk, NY, USA). Descriptive statistics and binary logistic regression analyses were carried out to describe the variables and to identify factors associated with HBV infection, respectively. Odds ratios (ORs) and 95% confidence intervals (CIs) were calculated, and p-values <0.05 were considered to indicate statistical significance.

### Ethical considerations

Ethical approval was obtained from the Department of Research and Ethical Review Committee of the Medical Laboratory Sciences, Addis Ababa University. Written consent was also obtained from the institutional review board of the College of Medicine and Health Sciences of Hawassa University and submitted to the ANC clinic of the hospital. Informed written consent was also obtained from the study participants for the collection of their sociodemographic data, the HIV results from their records, and to use their serum samples for HBsAg screening. A code number was used to ensure the confidentiality of the participants’ information. No participants paid for the tests that were performed and the results were given to the clinicians working in the ANC clinic on the same day for further diagnosis and management.

## RESULTS

### Sociodemographic characteristics

A total of 269 women attending the ANC clinic of the Hawassa University referral hospital from April to May, 2015 were enrolled in this study. The mean (standard deviation) age of the study participants was 26.0±4.5 years (range, 18-39 years), and a plurality (39.4%) were in the age category of 26 to 30 years. The majority of the study participants lived in urban areas (84.0%), a plurality were housewives (39.4%, n=106), and almost one-fourth of these women had an education level of less than secondary school (Table 1).

### Prevalence of HBV infection

Using the ELISA test as a reference, the overall seroprevalence of HBsAg was 7.8% (n=21). However, four of those samples were found to be negative by the rapid HBsAg test. As shown in Table 1, the study participants with no formal education were more likely to be infected (OR, 3.68; 95% CI, 1.27 to 10.68; p<0.05) than those who had completed secondary school. However, none of the other sociodemographic variables showed a significant association with HBV infection (Table 1).

### Risk factors for HBV infection

A total of 202 (75.1%) participants had been circumcised early in life, while 91 (33.8%) had been hospitalized at some time during their lives, 74 (27.5%) had a past history of abortion, 57 (21.2%) had undergone surgical procedures, 49 (18.2%) had a history of tattooing, and 39 (14.5%) had a history of multiple sexual partners. Only nine (3.3%) had received blood products, all of whom were negative for HBV. None of the respondents were aware of their HBV status or had been immunized against HBV. However, all of the study participants were screened for HIV during their ANC visit. The overall HIV seroprevalence rate was 5.2% (n=14), of whom nine (64.3%) were on antiretroviral therapy. Two of the HIV-positive participants (14.2%) were also positive for HBsAg, giving a 0.74% HIV/HBV co-infection rate. Although the difference was not statistically significant, HIV-positive women had higher odds of HBV infection (OR, 2.07; 95%CI, 0.43 to 9.93; p=0.36) than their counterparts. Of the study participants, 25 (9.3%) had no potential risks for exposure, whereas 36.1% had one type of exposure and 54.6% had a history of multiple exposures (range, 2 to 5 risk factors). Neither the type of potential risk factors nor multiple exposure status was significantly associated with HBV infection (Table 2).

## DISCUSSION

In highly endemic areas, antenatal HBV screening is of paramount importance for health planners and program managers to develop prevention strategies in populations at risk for disease transmission. In this study, the overall seroprevalence of HBsAg in pregnant women was 7.8%. This shows a level of endemicity of HBV infection qualifying as almost high according to the criteria of the World Health Organization [1]. This prevalence rate is higher than has been reported in previous studies in different regions of Ethiopia, such as 6.1% in a rural hospital in southern Ethiopia [7], 3% in Addis Ababa [9], 3.7% in Jimma [5], 5.3% in Debre-Tabor Hospital [6], and 3.8% in Bahir Dar city [8], Ethiopia. However, comparable or higher results have been reported in other African countries, such as 8.0% in Mali [16], 8.3% in Nigeria [17], 9.3% in Kenya [18], 9.5% in Gabon [19], 10.2% in Cameroon [20], and 12.6% in Ghana [21]. Variations in prevalence both within and outside of Ethiopia may be due to differences in the methods used for screening for HBsAg, geographical variations, and cultural and behavioral differences regarding possible risk factors of HBV infection. In developed nations, where regular screening and vaccination for HBV are performed, low prevalence rates (<2%) have been reported in pregnant women, such as 0.14% to 0.97%in the US (excluding Asian-Americans) [22], 0.9% in Brazil [23], 1.65% in Mexico [24], and1.6% in Saudi Arabia [25]. Similar practices in developing countries may reduce mother-to-child transmission and the public health significance of HBV infections.

Study participants with no formal education had higher odds of HBV infection than those who had completed at least secondary school. Other studies have also noted that the seroprevalence of HBV infection decreased with increasing levels of education [5,11,17]. This may be due to differences in the level of awareness about the transmission of HBV. In this study, age was found to have no significant associations with HBV infection, in agreement with some studies conducted elsewhere [8,9,26,27]. However, other studies have reported that HBV infection in pregnant women increased with age [24] and a significantly higher prevalence was observed in women between the ages of 20 to 24 years [5,28]. Moreover, a higher rate of HBV was observed among daily laborers and students, in contrast to women with regular employment, but this finding was not statistically significant.

Of the 14 women who were HIV-positive, only two women tested positive for HBsAg. Although this finding was not statistically significant, HIV-positive women had a higher likelihood of HBV infection than their counterparts, in agreement with other studies [9,29]. In contrast to our study, previous study in Cameroon [20] have found that HIV infection was closely associated with HBV infection. In this study, the HIV/HBV co-infection rate was 0.74%. This finding is comparable with previous studies conducted in a rural hospital in southern Ethiopia (0.6%) [7] and among urban pregnant women in Yaoundé (0.74%) [30]. However, this rate is lower than co-infection rates that have been reported in northwest Ethiopia (1.3%) [8] and Cameroon (1.5%) [20], and significantly lower than the rate that has been reported in Nigeria (4.2%) [17]. Differences in the co-infection rate may be due to 64.3% of HIV-positive women in our study were on antiretroviral therapy, which may reduce the replication of HBV-DNA levels that ultimately decreases viral antigen below the detection limit (occult hepatitis) and probably increase spontaneous clearance of the virus.

In this study, neither the type of potential risk factors nor multiple exposure status was significantly associated with HBV infection, in accordance with a previous study conducted in Addis Ababa [9]. However, other studies in Ethiopia and African countries have reported that blood transfusion [8,20]. body tattooing [6,8,17,27], circumcision [27], and surgery [6,8,20] were potential risk factors for HBV infection. Likewise, HBsAg positivity was previously reported to be significantly higher in pregnant women who had a previous history of abortion [5,28]. However, no significant association between previous history of abortion and HBsAg positivity was observed in our study. This may be due to the implementation of policies aimed at reducing the incidence of unsafe abortions, health education related to HIV, and the promotion of the use of barriers as contraception.

Nevertheless, the absence of associations between various risk factors and HBV infection may have been due to the small sample size. Moreover, as a hospital-based study that used a non-probabilistic sampling method, selection bias may be introduced, potentially hindering the generalizability of our results to all pregnant women in the study area.

In summary, a high prevalence of HBV infection was detected among pregnant women in the study area. Study participants with no formal education had higher odds of infection than those with at least a secondary-school education. Neither the type of potential risk factors nor multiple exposure status was significantly associated with HBV infection. Screening of pregnant women for HBV regardless of risk factors and increasing awareness regarding transmission and prevention may reduce the risk of infections. This finding provides insights for policy makers and concerned bodies about screening pregnant women for HBV during their ANC follow-up visits. During data collection, those who were HBsAg-positive were referred to the attending physician, but it was not possible to find supplies for immunoglobulin prophylaxis either in the hospital pharmacy or in the town. Therefore, our findings may provide an impetus for suppliers to start providing immunoglobulin to vaccinate newborns immediately after delivery.

## Figures and Tables

**Table 1.** Seroprevalence of HBV and sociodemographic characteristics of pregnant women attending antenatal care at the Hawassa University referral hospital from April to May, 2015

Variable	Total	HBV staus of mothers			
		Positive	Negative	Crude OR (95% CI)	p-value
Age (yr)					
18-20	43 (16.0)	3 (7.0)	40 (93.0)	1.00 (reference)	
21-25	84 (31.2)	6 (7.1)	78 (92.9)	1.03 (0.24, 4.32)	0.97
26-30	106 (39.4)	9 (8.5)	97 (91.5)	1.24 (0.32, 4.81)	0.76
31-39	36 (13.4)	3 (8.3)	33 (91.7)	1.21 (0.23, 6.41)	0.82
Residence					
Urban	226 (84.0)	16 (7.1)	210 (92.9)	1.00 (reference)	
Rural	43 (16.0)	5 (11.6)	38 (88.4)	1.73 (0.60, 4.99)	0.31
Educational status					
No formal education	31 (11.5)	6 (19.4)	25 (80.6)	3.68 (1.27, 10.68)	0.02
Primary	42 (15.6)	3 (7.1)	39 (92.9)	1.18 (0.32, 4.38)	0.80
Secondary or above	196 (72.8)	12 (6.1)	184 (93.9)	1.00 (reference)	
Occupation					
Employed	99 (36.8)	4 (4.0)	95 (96.0)	1.00 (reference)	
Housewife	106 (39.4)	10 (9.4)	96 (90.6)	2.47 (0.75, 8.16)	0.14
Daily laborer	6 (2.2)	1 (16.7)	5 (83.3)	4.75 (0.44, 50.74)	0.20
Merchant	38 (14.1)	3 (7.9)	35 (92.1)	2.04 (0.43, 9.56)	0.37
Student	20 (7.4)	3 (15.4)	17 (85.0)	4.19 (0.86, 20.42)	0.08
Gestational age					
1st trimester	87 (32.3)	10 (11.5)	77 (88.5)	2.52 (0.83, 7.68)	0.10
2nd trimester	80 (29.7)	6 (7.5)	74 (92.5)	1.57 (0.46, 5.35)	0.47
3rd trimester	102 (37.9)	5 (4.9)	97 (95.1)	1.00 (reference)	
Previous place of delivery					
No birth	109 (40.5)	6 (5.5)	103 (94.5)	1.00 (reference)	
Home	47 (17.5)	6 (12.8)	41 (87.2)	2.51 (0.77, 8.24)	0.13
Health institution	113 (42.0)	9 (8.0)	104 (92.0)	1.49 (0.51, 4.32)	0.47

Values are presented as number (%). HBV, hepatitis B virus; OR, odds ratio; CI, confidence interval.

**Table 2.** Risk factors and prevalence of HBsAg among pregnant women attending antenatal care at the Hawassa University referral hospital from April to May, 2015

Variable		HBV status of mothers (n= 269)				
		Total	Neg	Pos	Crude OR (95% CI)	p-value
History of blood transfusions	Yes	9 (3.3)	9 (100)	0 (0.0)	0.00	0.99
	No	260 (96.7)	239 (91.9)	21 (8.1)	1.00 (reference)	
Multiple sexual partners	Yes	39 (14.5)	37 (94.9)	2(5.1)	0.61 (0.13, 2.69)	0.09
	No	230 (85.5)	211 (91.7)	19 (8.3)	1.00 (reference)	
Hospital admissions	Yes	91 (33.8)	83 (91.2)	8 (8.8)	1.23 (0.49, 3.07)	0.67
	No	178 (66.2)	165 (92.7)	13 (7.3)	1.00 (reference)	
Circumcision	Yes	202 (75.1)	183 (90.6)	19 (9.4)	3.40 (0.76, 14.89)	0.11
	No	67 (24.9)	65 (97.0)	2 (3.0)	1.00 (reference)	
Surgical procedures	Yes	57 (21.2)	51 (89.5)	6 (10.5)	1.52 (0.57, 4.18)	0.39
	No	212 (78.8)	197 (92.9)	15 (7.1)	1.00 (reference)	
Body tattooing	Yes	49 (18.2)	46 (93.9)	3(6.1)	0.73 (0.21,2.59)	0.63
	No	220 (81.8)	202 (91.8)	18 (8.2)	1.00 (reference)	
History of abortion	Yes	74 (27.5)	67 (90.5)	7(9.5)	1.40 (0.52, 3.49)	0.53
	No	195 (72.5)	181 (92.8)	14 (7.2)	1.00 (reference)	
HIV/AIDS status	Neg	255 (94.8)	236 (92.5)	19 (7.5)	1.00 (reference)	0.36
	Pos	14 (5.2)	12 (85.7)	2 (14.3)	2.07 (0.43, 9.93)	
Risk exposure status	No	25 (9.3)	24 (96.0)	1 (4.0)	1.00 (reference)	
	1	97 (36.1)	90 (92.8)	7(7.2)	1.87 (0.22, 15.91)	0.57
	≥2	147 (54.6)	134 (91.2)	13 (8.8)	2.33 (0.29-18.63)	0.43

Values are presented as number (%). HBV, hepatitis B virus; HBsAg, hepatitis B surface antigen; Neg, negative; Pos, positive; OR, odds ratio; CI, confidence interval. HIV, human immunodeficiency virus; AIDS, acquired immune deficiency syndrome.
